# Trends in Telehealth Visits During Pregnancy, 2018 to 2021

**DOI:** 10.1001/jamanetworkopen.2023.6630

**Published:** 2023-04-04

**Authors:** Mahip Acharya, Mir M. Ali, Corey J. Hayes, Cari A. Bogulski, Everett F. Magann, Hari Eswaran

**Affiliations:** 1 Institute for Digital Health & Innovation, University of Arkansas for Medical Sciences, Little Rock; 2 Department of Biomedical Informatics, University of Arkansas for Medical Sciences, Little Rock; 3 Center for Mental Healthcare and Outcomes Research, Central Arkansas Veterans Healthcare Systems, North Little Rock; 4 Department of Obstetrics and Gynecology, University of Arkansas for Medical Sciences, Little Rock

## Abstract

This cross-sectional study examines trends of prenatal telehealth visits in pregnancy and explores patient characteristics associated with the number of prenatal telehealth visits.

## Introduction

The COVID-19 pandemic has affected prenatal care practice, with an increase in the adoption of alternative care models.^1^ Telehealth visits are useful for quick patient follow-ups but are of limited value when laboratory tests or ultrasonography are required.^1^ We assessed trends of prenatal telehealth visits in pregnancy and explored patient characteristics associated with the number of prenatal telehealth visits.

## Methods

This cross-sectional study used IQVIA PharMetrics Plus for Academics data (2018-2021), a health insurance claims database representative of commercially insured individuals in the United States. We identified deliveries by individuals aged 15 to 50 years between September 2018 and October 2021 using diagnosis and procedure codes (eTable in Supplement 1). The 40 weeks (280 days) prior to delivery were considered the baseline period, based on the estimated length of a full-term pregnancy. We required continuous enrollment during the baseline period. Pregnancies were classified as high-risk if the maternal age at delivery was older than 35 years or if certain clinical characteristics were present (eTable in Supplement 1). The study was determined not human participant research by University of Arkansas for Medical Sciences institutional review board, as the data used were fully deidentified; therefore, the requirement for informed consent was waived. We followed the STROBE reporting guideline for cross-sectional studies.

Prenatal telehealth visits were identified during the baseline period using a combination of place of service, procedure codes, physician specialty, and pregnancy supervision diagnosis codes (eTable in Supplement 1). We studied trends in prenatal telehealth utilization during the prior 40 weeks for all deliveries by delivery month. A pregnancy was recorded as having a telehealth visit if at least 1 prenatal telehealth visit was identified in the 40-week period. We also restricted samples to high-risk pregnancies and performed similar trend analyses. Furthermore, we performed trend analyses where pregnancies, prenatal telehealth, and insurance coverage were assessed on a month-by-month basis: all active pregnancies each month were used as denominators and number of pregnancies with at least 1 telehealth visit during that month as numerators. Multivariable Poisson regressions were estimated to examine associations between demographic and clinical characteristics and the number of telehealth visits for deliveries in September 2018 to February 2020 (prepandemic period) and March 2020 to October 2021 (pandemic period), separately for all and high-risk pregnancies. All analyses were conducted using SAS version 9.4 (SAS Institute).

## Results

There were 45 203 pregnancy episodes identified. The mean (SD) age was 31.5 (5.2) years, 5549 (12.3%) had gestational diabetes, and 26 485 (58.6%) had a high-risk pregnancy. Prenatal telehealth use during 40-week pregnancy by month of delivery was: 1.3% (September 2018), 1.1% (January 2020), 17.3% (November 2020), and 9.9% (October 2021). Monthly telehealth utilization rates for all active pregnancies were: 0.1% (September 2018), 0.1% (January 2020), 2.8% (April 2020), and 0.5% (October 2021). High-risk pregnancies had slightly higher telehealth use in both episodic and monthly approaches (Figure).

**Figure.**
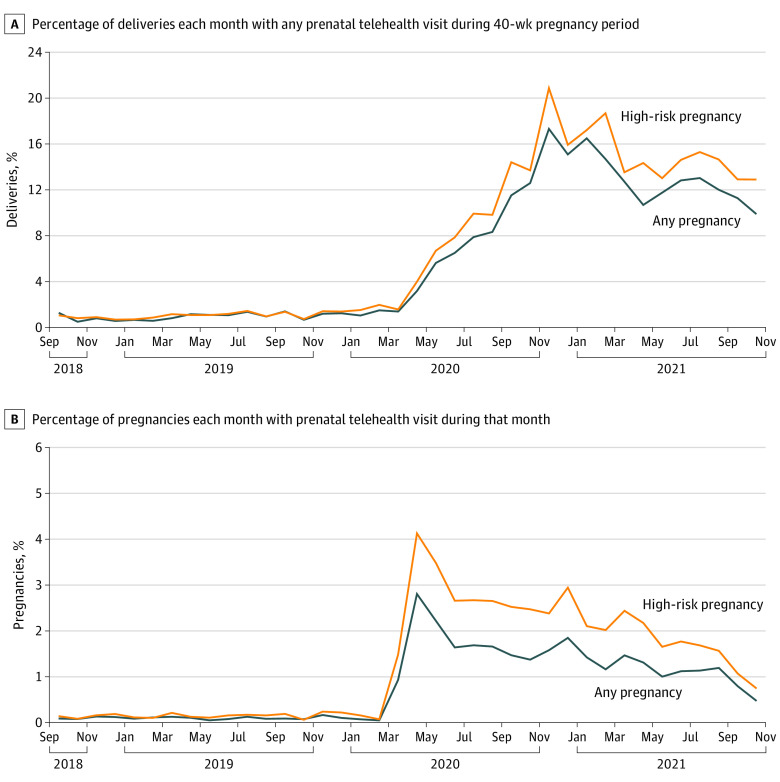
Trends in Any Prenatal Telehealth Visit During 40-Week Pregnancy Period for All Pregnancies and High-risk Pregnancies There was a total of 45 203 pregnancies and 26 485 high-risk pregnancies in the sample.

Multivariable regressions for all deliveries during the pandemic showed incidence rate ratios (IRRs) of 1.38 (95% CI, 1.23-1.54) and 1.77 (95% CI, 1.61-1.94) for participants with anxiety and depression, respectively. Patients receiving Medicaid had 165% higher prenatal telehealth rates for deliveries in the pandemic period. There was no statistically significant association in the prepandemic period, although the point estimate indicated much lower telehealth use. Similar results were observed for high-risk pregnancies (Table).

**Table.** Multivariable Poisson Regression Results for Total Number of Prenatal Telehealth Visits Across Patient Characteristics for Deliveries Occurring September 2018 to February 2020 (Prepandemic Period) and March 2020 to October 2021 (Pandemic Period)

Characteristics	IRR (95% CI)			
	Prepandemic (Sep 2018 to Feb 2020)		Pandemic (Mar 2020 to Oct 2021)	
	All pregnancies (n = 26 365)	High-risk pregnancies (n = 15 497)	All pregnancies (n = 18 838)	High-risk pregnancies (n = 10 988)
Age, y	1.04 (1.02-1.06)	1.03 (1.00-1.05)	1.03 (1.02-1.04)	1.01 (1.00-1.02)
Region of residence				
East	1 [Reference]	1 [Reference]	1 [Reference]	1 [Reference]
Midwest	0.55 (0.33-0.94)	0.61 (0.34-1.10)	0.76 (0.68-0.85)	0.73 (0.64-0.83)
South	3.32 (2.22-4.97)	2.79 (1.80-4.31)	0.48 (0.42-0.55)	0.45 (0.39-0.52)
West	0.58 (0.22-1.79)	1.33 (0.81-2.16)	1.13 (1.02-1.27)	1.11 (0.98-1.25)
Missing	NA	NA	0.88 (0.45-1.69)	0.99 (0.49-2.00)
Insurance type				
Commercial	1 [Reference]	1 [Reference]	1 [Reference]	1 [Reference]
Medicaid	0.16 (0.02-1.18)	0.23 (0.03-1.68)	2.65 (2.32-3.02)	2.47 (2.09-2.92)
Others/missing	NA	NA	1.37 (1.06-1.76)	1.32 (0.99-1.76)
Clinical diagnoses				
Gestational diabetes	1.24 (0.92-1.66)	1.21 (0.86-1.69)	3.49 (3.24-3.76)	3.27 (3.00-3.56)
Major depressive disorder	0.99 (0.59-1.68)	0.84 (0.45-1.57)	1.38 (1.23-1.54)	1.33 (1.17-1.52)
Anxiety disorders	1.06 (0.70-1.60)	1.17 (0.74-1.86)	1.77 (1.61-1.94)	1.73 (1.55-1.93)
Nicotine dependence	0.95 (0.55-1.63)	0.92 (0.48-1.74)	1.20 (1.04-1.37)	1.25 (1.08-1.46)
Alcohol use disorder	NA	NA	1.08 (0.70-1.68)	0.77 (0.43-1.37)
Other substance use disorder	1.16 (0.42-3.15)	1.11 (0.35-3.53)	0.83 (0.64-1.07)	0.98 (0.73-1.32)

Abbreviations: IRR, incidence risk ratio; NA, not applicable (coefficients not estimated due to zero or extremely small cell counts).

## Discussion

Prenatal telehealth visits increased substantially during the early phase of the COVID-19 pandemic: the highest percentage of pregnancies with telehealth was observed in April 2020. Deliveries in November 2020 had the highest telehealth visit rates during the 40-week pregnancy. Previous studies have reported similar rates of telehealth encounters during pregnancy for patients with high and low risk as well as those with Medicaid and commercial insurance.^2,3^ Higher telehealth rates among patients with Medicaid are likely explained by state-level policy changes in telehealth coverage and reimbursement.^4^ Higher telehealth utilization among pregnant patients with anxiety and depression aligns with overall telehealth use patterns during the pandemic.^5^ Alternative prenatal care models have been proposed that incorporate virtual visits for mental health screening.^6^ This study’s limitations include potential lack of generalizability to pregnancies covered by Medicaid, as our sample comprises almost exclusively commercially insured patients; our inability to assess clinicians’ and patients’ preferences; and inability to evaluate whether telehealth was a substitution for or addition to in-person visits.

## Notes

Supplement 1. **eTable. **Identification of Telehealth, Pregnancy Supervision, Deliveries, and High-risk Conditions
eReferences.

Supplement 2.
Data Sharing Statement
