# Supplementary material for: Trends in Telehealth Visits During Pregnancy, 2018 to 2021
Source: JAMA Netw Open. 2023 Apr 4;6(4):e236630. doi: 10.1001/jamanetworkopen.2023.6630 (PMC10074218; doi:10.1001/jamanetworkopen.2023.6630)
Supplement: Supplement 1. — eTable. Identification of Telehealth, Pregnancy Supervision, Deliveries, and High-risk Conditions eReferences. [file jamanetwopen-e236630-s001.pdf]

Supplemental Online Content

Acharya M, Ali MM, Hayes CJ, Bogulski CA, Magann EF, Eswaran H. Trends in telehealth visits during pregnancy, 2018 to 2021. *JAMA Netw Open.* 2023;6(4):e236630. doi:10.1001/jamanetworkopen.2023.6630

eTable. Identification of Telehealth, Pregnancy Supervision, Deliveries, and High-risk Conditions

eReferences.

This supplemental material has been provided by the authors to give readers additional information about their work.

© 2023 Acharya M et al. *JAMA Network Open.*

eTable. Identification of Telehealth, Pregnancy Supervision, Deliveries, and High-risk Conditions

Characteristics	Claims-based identification
Telehealth[1]	Place of service: '2', OR procedure codes: '99441', '99442', '99443', OR modifiers: '95', 'GT', 'GQ', 'G0'
Pregnancy supervision	**ICD-10-CM:** 'Z34', 'O00', 'O01', 'O02', 'O03', 'O04', 'O05', 'O06', 'O07', 'O08', 'O09', 'O10', 'O11', 'O12', 'O13', 'O14', 'O15', 'O16', 'O20', 'O21', 'O22', 'O23', 'O24', 'O25', 'O26', 'O27', 'O28', 'O29', 'O30', 'O31', 'O32', 'O33', 'O34', 'O35', 'O36', 'O37', 'O38', 'O39', 'O40', 'O41', 'O42', 'O43', 'O44', 'O45', 'O46', 'O47', 'O48', 'O60', 'O61', 'O62', 'O63', 'O64', 'O65', 'O66', 'O67', 'O68', 'O69', 'O70', 'O71', 'O72', 'O73', 'O74', 'O75', 'O76', 'O77', 'O80', 'O81', 'O82', 'O85', 'O86', 'O87', 'O88', 'O89', 'O90', 'O91', 'O92', 'O94', 'O98', 'O99', 'O9A'
Prenatal care-related telehealth visits	(Telehealth visit AND physician specialty: OB/GYN, Midwife, General Practice/Family Physician) OR (Telehealth visit AND physician specialty: Nurse Practitioner, Clinical Social Worker, Unknown/Missing/Other AND pregnancy supervision codes)
End-of-pregnancy/deliveries	**ICD-10-CM**: 'O1002', 'O1012', 'O1022', 'O1032', 'O1042', 'O1092', 'O114', 'O1204', 'O1214', 'O1224', 'O134', 'O1404', 'O1414', 'O1424', 'O1494', 'O151', 'O164', 'O2402', 'O2412', 'O2432', 'O24420', 'O24424', 'O24425', 'O24429', 'O2482', 'O2492', 'O252', 'O6010X0', 'O6010X1', 'O6010X2', 'O6010X3', 'O6010X4', 'O6010X5', 'O6010X9', 'O6012X0', 'O6012X1', 'O6012X2', 'O6012X3', 'O6012X4', 'O6012X5', 'O6012X9', 'O6013X0', 'O6013X1', 'O6013X2', 'O6013X3', 'O6013X4', 'O6013X5', 'O6013X9', 'O6014X0', 'O6014X1', 'O6014X2', 'O6014X3', 'O6014X4', 'O6014X5', 'O6014X9', 'O6020X0', 'O6020X1', 'O6020X2', 'O6020X3', 'O6020X4', 'O6020X5', 'O6020X9', 'O6022X0', 'O6022X1', 'O6022X2', 'O6022X3', 'O6022X4', 'O6022X5', 'O6022X9', 'O6023X0', 'O6023X1', 'O6023X2', 'O6023X3', 'O6023X4', 'O6023X5', 'O6023X9', 'O620', 'O621', 'O622', 'O623', 'O624', 'O628', 'O629', 'O630', 'O631', 'O632', 'O639', 'O640XX0', 'O640XX1', 'O640XX2', 'O640XX3', 'O640XX4', 'O640XX5', 'O640XX9', 'O641XX0', 'O641XX1', 'O641XX2', 'O641XX3', 'O641XX4', 'O641XX5', 'O641XX9', 'O642XX0', 'O642XX1', 'O642XX2', 'O642XX3', 'O642XX4', 'O642XX5', 'O642XX9', 'O643XX0', 'O643XX1', 'O643XX2','O643XX3', 'O643XX4', 'O643XX5', 'O643XX9', 'O644XX0', 'O644XX1', 'O644XX2', 'O644XX3', 'O644XX4', 'O644XX5', 'O644XX9', 'O645XX0', 'O645XX1', 'O645XX2', 'O645XX3', 'O645XX4', 'O645XX5', 'O645XX9', 'O648XX0', 'O648XX1', 'O648XX2', 'O648XX3', 'O648XX4', 'O648XX5', 'O648XX9', 'O649XX0', 'O649XX1', 'O649XX2', 'O649XX3', 'O649XX4', 'O649XX5', 'O649XX9', 'O650', 'O651', 'O652', 'O653', 'O654', 'O655', 'O658', 'O659', 'O660', 'O661', 'O662', 'O663', 'O6640', 'O6641', 'O665', 'O666', 'O668', 'O669', 'O670', 'O678', 'O679', 'O68', 'O690XX0', 'O690XX1', 'O690XX2', 'O690XX3', 'O690XX4',

© 2023 Acharya M et al. *JAMA Network Open.*

	'O690XX5', 'O690XX9', 'O691XX0', 'O691XX1', 'O691XX2', 'O691XX3', 'O691XX4', 'O691XX5', 'O691XX9', 'O692XX0', 'O692XX1', 'O692XX2', 'O692XX3', 'O692XX4', 'O692XX5', 'O692XX9', 'O693XX0', 'O693XX1', 'O693XX2', 'O693XX3', 'O693XX4', 'O693XX5', 'O693XX9', 'O694XX0', 'O694XX1', 'O694XX2', 'O694XX3', 'O694XX4', 'O694XX5', 'O694XX9', 'O695XX0', 'O695XX1', 'O695XX2', 'O695XX3', 'O695XX4', 'O695XX5', 'O695XX9', 'O6981X0', 'O6981X1', 'O6981X2', 'O6981X3', 'O6981X4', 'O6981X5', 'O6981X9', 'O6982X0', 'O6982X1', 'O6982X2', 'O6982X3', 'O6982X4', 'O6982X5', 'O6982X9', 'O6989X0', 'O6989X1', 'O6989X2', 'O6989X3', 'O6989X4', 'O6989X5', 'O6989X9', 'O699XX0', 'O699XX1', 'O699XX2', 'O699XX3', 'O699XX4', 'O699XX5', 'O699XX9', 'O700', 'O701', 'O702', 'O7020', 'O7021', 'O7022', 'O7023', 'O703', 'O704', 'O709', 'O711', 'O712', 'O713', 'O714', 'O715', 'O716', 'O717', 'O7181', 'O7182', 'O7189', 'O719', 'O740', 'O741', 'O742', 'O743', 'O744', 'O745', 'O746', 'O747', 'O748', 'O749', 'O750', 'O751', 'O752', 'O753', 'O754', 'O755', 'O7581', 'O7582', 'O7589', 'O759', 'O770', 'O771', 'O778', 'O779', 'O80', 'O82', 'O8802', 'O8812', 'O8822', 'O8832', 'O8882', 'O9802', 'O9812', 'O9822', 'O9842', 'O9852', 'O9862', 'O9872', 'O9882', 'O9892', 'O9902', 'O9912', 'O9952', 'O9962', 'O9972', 'O99814', 'O99844', 'O9A12', 'O9A22', 'O9A32', 'O9A42', 'O9A52', 'Z370', 'Z371', 'Z372', 'Z373', 'Z374', 'Z3750', 'Z3751', 'Z3752', 'Z3753', 'Z3754', 'Z3759', 'Z3760', 'Z3761', 'Z3762', 'Z3763', 'Z3764', 'Z3769', 'Z377', 'Z379', 'O2662', 'O2672', 'O9832', 'O99214', 'O99284', 'O99314', 'O99324', 'O99334', 'O99344', 'O99354', 'O9942', 'O99824', 'O99834', 'O99892', 'Z3800', 'Z3801', 'Z381', 'Z382', 'Z3830', 'Z3831', 'Z384', 'Z385', 'Z3861', 'Z3862', 'Z3863', 'Z3864', 'Z3865', 'Z3866', 'Z3868', 'Z3869', 'Z387', 'Z388'<br><br>**ICD-9-CM**: Gathered from ICD-10-CM to ICD-9-CM conversion algorithm[2]<br><br>**ICD-10-PCS**: '3E0P3VZ', '3E0P7VZ', '3E0DXGC', '10907ZA', '10908ZA', '10E0XZZ', '10D00Z0', '10D00Z1', '10D00Z2', '10900ZC', '10903ZC', '10904ZC', '10907ZC', '10908ZC', '10D07Z3', '10D07Z4', '10D07Z5', '10D07Z6', '10D07Z7', '10D07Z8', '10S07ZZ','10S0XZZ', '0W8NXZZ', '0U7C7ZZ', '0Q820ZZ', '0Q823ZZ', '0Q824ZZ', '0Q830ZZ', '0Q833ZZ', '0Q834ZZ', '10J07ZZ', '3E030VJ', '3E033VJ', '3E040VJ', '3E043VJ', '3E050VJ', '3E053VJ', '3E060VJ', '3E063VJ', '3E040VG', '3E040VH', '3E013VG', '3E013VJ', '3E030VG', '3E030VH', '3E033VG', '3E033VH', '3E043VG', '3E043VH', '3E050VG', '3E050VH', '3E053VG', '3E053VH', '3E060VG', '3E060VH', '3E063VG', '3E063VH', '10A00ZZ', '10A03ZZ', '10A04ZZ', '10A07ZZ', '10A08ZZ', '10A07Z6', '10A07ZW', '10A07ZX', '10T20ZZ', '10T23ZZ', '10T24ZZ', '10D20ZZ', '10D24ZZ', '10D27ZZ', '10D28ZZ', '10J20ZZ', '10J23ZZ', '10J24ZZ', '10J27ZZ',

© 2023 Acharya M et al. *JAMA Network Open.*

	'10J28ZZ', '10J2XZZ', '10S20ZZ', '10S23ZZ', '10S24ZZ', '10S27ZZ', '10S28ZZ', '10T27ZZ' , '10T28ZZ'<br><br>**ICD-9-PCS**: Gathered using conversion algorithm[1]<br><br>**CPT-4**: '59400’, '59409’, '59410’, '59412, '59414’, '59510’, '59514’, '59525’, '59610’, '59612’, '59614’, '59618’, '59620’, '59622’, '59899’
**High risk conditions (selected based on the criteria used by University of Arkansas for Medical Sciences High-Risk Pregnancy Program)**	
Severe asthma	**ICD-9-CM**: '493'<br>**ICD-10-CM**: 'J45'
Cardiac disease	**ICD-9-CM**: '390', '391', '392', '393', '394', '395', '396', '397', '398', '399', '410', '414', '416', '417', '424', '425', '426', '427', '428', '429', '6486'<br>**ICD-10-CM**: 'I00', 'I01', 'I02', 'I05', 'I06', 'I07', 'I08', 'I09', 'I21', 'I22', 'I23', 'I25', 'I27', 'I28', 'I34', 'I35', 'I36', 'I37', 'I38', 'I39', 'I42', 'I44', 'I45', 'I46', 'I47', 'I48', 'I49', 'I50', 'I51', 'O994', 'I970', 'I971'
Pre-existing diabetes	**ICD-9-CM**: '250', '6480', '2714'<br>**ICD-10-CM**: 'E10', 'O240', 'E11', 'O241', 'E13', 'O243', 'O248', 'O249'
Pre-existing hypertension	**ICD-9-CM**: '401', '402', '403', '404', '405', '6420', '6421', '6422'<br>**ICD-10-CM**: 'I10', 'I11', 'I12', 'I13', 'I14', 'I15', 'I16', 'O10', 'O11'
Epilepsy	**ICD-9-CM**: '345', '78039'<br>**ICD-10-CM**: 'G40'
Hemolytic anemia	**ICD-9-CM**: '282'<br>**ICD-10-CM**: 'D55', 'D56', 'D57', 'D58'
Pulmonary embolism	**ICD-9-CM**: '673', '6712', '6713'<br>**ICD-10-CM**: 'O88', 'O222', 'O223'
Other pulmonary disease	**ICD-9-CM**: '492', '500', '501', '502', '503', '504', '505', '506', '507', '508', '509', '510', '511', '512', '513', '514', '515', '516', '517', '518', '519', '494', '010', '011', '012', '013', '014', '015', '016', '017', '018', '481', '482', '483', '484', '485', '486', '415', '416', '417', '496', '1363', '6473', 'V461'<br>**ICD-10-CM**: 'A078', 'A221', 'A3791', 'A481', 'B250', 'B440', 'J13', 'J14', 'J15', 'J16', 'J17', 'J18', 'J43', 'J44', 'J47', 'B4481', 'J60', 'J61', 'J62', 'J63', 'J64', 'J65', 'J66', 'J68', 'J69', 'J70', 'J80', 'J81', 'J82', 'J83', 'J84', 'J85', 'J86', 'J90', 'J91', 'J92', 'J93', 'J94', 'J96', 'J97', 'J98', 'J99', 'J950', 'J951', 'J952', 'J953', 'J9581', 'J9582', 'J9584', 'A15', 'A16', 'A17', 'A18', 'A19', 'I26', 'I27', 'I28', 'B59', 'O980', 'Z991'
Renal disease	**ICD-9-CM**: '582', '583', '584', '585', '586', '587', '588', '592', '6462'<br>**ICD-10-CM**: 'N03', 'N05', 'N06', 'N07', 'N08', 'N14', 'N15', 'N16', 'N17', 'N18', 'N19', 'N25', 'N261', 'N269', 'O2683', 'O121', 'O122'
Coagulation disorders	**ICD-9-CM**: '286'<br>**ICD-10-CM**: 'D65', 'D66', 'D67', 'D68'
Cranial injury	**ICD-9-CM**: '430', '431', '432', '433', '434', '4370', '4371', '4372', '4373', '4374', '4375', '4376'<br>**ICD-10-CM**: 'I60', 'I61', 'I62', 'I63', 'I64', 'I65', 'I66', 'I671', 'I672', 'I674', 'I675', 'I676', 'I677', 'I6781', 'I6782'
HIV/AIDS	**ICD-9-CM**: '042', 'V08'

© 2023 Acharya M et al. *JAMA Network Open.*

	**ICD-10-CM**: 'B20', 'Z21'
Cervical cancer	**ICD-9-CM**: '2331', '180', '181', '182', '183', '184', '6221'<br>**ICD-10-CM**: 'D06', 'C53', 'C58', 'C54', 'C56', 'C57', 'C51', 'C52', 'N87'
Other systemic disease	**ICD-9-CM**: '242', '243', '244', '038', '174', '070', '6481', '6954', '7100', '575', '5770', '5771', '5714', '6474', '6467'<br>**ICD-10-CM**: 'E05', 'E00', 'E018', 'E030', 'E031', 'E032', 'E033', 'E038', 'E039', 'E890', 'A40', 'A41', 'C5001', 'C5011', 'C5021', 'C5031', 'C5041', 'C5051', 'C5061', 'C5081', 'C5091', 'B15', 'B16', 'B17', 'B18', 'B19', 'O9928', 'L93', 'M32', 'K81', 'K82', 'K85', 'K860', 'K861', 'K73', 'K754', 'O266'
Incompetent cervix	**ICD-9-CM**: '6545'<br>**ICD-10-CM**: 'O343'
Pre-eclampsia/Eclampsia	**ICD-9-CM**: '6425', '6426', '6427'<br>**ICD-10-CM**: 'O141', 'O142', 'O15', 'O11'
Suspected fetal abnormalities	**ICD-9-CM**: '6550', '6551', '6552', '6553', '6554', '6555', '65560', '65561', '65562', '65563', '6558', '65590', '65591', '65592', '65593'<br>**ICD-10-CM**: O350', 'O351', 'O352', 'O353', 'O354', 'O355', 'O356', 'O358', 'O359'
Polyhydramnios	**ICD-9-CM**: '657'<br>**ICD-10-CM**: 'O401', 'O402'
Oligohydramnios	**ICD-9-CM**: '658'<br>**ICD-10-CM**: 'O410'
Premature rupture of membrane	**ICD-9-CM**: '6581', '6582', '6583'<br>**ICD-10-CM**: 'O4201', 'O4211', 'O4291'
Hyperemesis	**ICD-9-CM**: '6432'<br>**ICD-10-CM**: 'O212'
Placenta previa	**ICD-9-CM**: '641'<br>**ICD-10-CM**: 'O44', 'O45', 'O46'
Birth plurality	**ICD-9-CM**: '6510', '6513', 'V31', 'V32', 'V33', 'V34', 'V35', 'V36', 'V37', 'V272', 'V273', 'V274', '6511', '6512', '6514', '6515', '6516', '6517', '6518', '6519', 'V275', 'V276', 'V277'<br>**ICD-10-CM**: 'O3001', 'O3002', 'O3003', 'O301', 'O302', 'Z375', 'Z376', 'Z377'
**Conditions used in the regression analyses**	
Gestational diabetes	**ICD-9-CM**: '6488', '7902'<br>**ICD-10-CM**: 'O244', 'O9981', 'R73'
Nicotine dependence	**ICD-9-CM**: '3051', 'V1582'<br>**ICD-10-CM**: 'F17200', 'Z87891'
Alcohol use disorder	**ICD-9-CM**: '303'<br>**ICD-10-CM**: 'F10'
Other substance use disorder	**ICD-9-CM**: '2910', '2911', '2912', '2913', '2914', '2915', '29181', '29182', '29189', '2919', '30500', '30501', '30502', '30503', '2920', '29211', '29212', '2922', '29281', '29282', '29283', '29284', '29285', '29289', '2929', '30410', '30411', '30412', '30413', '30420', '30421', '30422', '30423', '30430', '30431', '30432', '30433', '30440', '30441',

	'30442', '30443', '30450', '30451', '30452', '30453', '30460', '30461', '30462', '30463', '30480', '30481', '30482', '30483', '30490', '30491', '30492', '30493', '30520', '30521', '30522', '30523', '30530', '30531', '30532', '30533', '30540', '30541', '30542', '30543', '30570', '30571', '30572', '30573', '30580', '30581', '30582', '30583', '30590', '30591', '30592', '30593', '64830', '64833', '64834', '76075', '7795', 'V6542'<br>**ICD-10-CM**: 'F11159', 'F11181', 'F11182', 'F11188', 'F11222', 'F11259', 'F11281', 'F11282', 'F11288', 'F11922', 'F11959', 'F11981', 'F11982', 'F11988', 'F1210', 'F12122', 'F12159', 'F12180', 'F12188', 'F1220', 'F1221', 'F12222', 'F12259', 'F12280', 'F12288', 'F1290', 'F12922', 'F12959', 'F12980', 'F12988', 'F1310', 'F13159', 'F13180', 'F13181', 'F13182', 'F13188', 'F1320', 'F1321', 'F13259', 'F13280', 'F13281', 'F13282', 'F13288', 'F13959', 'F13980', 'F13981', 'F13982', 'F13988', 'F14122', 'F14159', 'F14180', 'F14181', 'F14182', 'F14188', 'F1420', 'F1421', 'F14222', 'F14259', 'F14280', 'F14281', 'F14282', 'F14288', 'F14922', 'F14959', 'F14980', 'F14981', 'F14982', 'F14988', 'F1510', 'F15122', 'F15159', 'F15180', 'F15181', 'F15182', 'F15188', 'F1520', 'F1521', 'F15222', 'F15259', 'F15280', 'F15281', 'F15282', 'F15288', 'F15920', 'F15922', 'F15959', 'F15980', 'F15981', 'F15982', 'F15988', 'F1610', 'F16122', 'F16159', 'F16180', 'F16183', 'F16188', 'F1620', 'F1621', 'F16259', 'F16280', 'F16283', 'F16288', 'F16959', 'F16980', 'F16983', 'F16988', 'F17208', 'F17218', 'F17228', 'F17298', 'F1810', 'F18159', 'F18180', 'F18188', 'F18259', 'F18280', 'F18288', 'F18959', 'F18980', 'F18988', 'F1910', 'F19122', 'F19159', 'F19180', 'F19181', 'F19182', 'F19188', 'F1920', 'F1921', 'F19222', 'F19259', 'F19280', 'F19281', 'F19282', 'F19288', 'F19921', 'F19922', 'F19939', 'F1994', 'F19950', 'F19951', 'F19959', 'F1996', 'F1997', 'F19980', 'F19981', 'F19982', 'F19988', 'F1999', 'O99320', 'O99321', 'O99322', 'O99323', 'O99325', 'P0441', 'P961', 'P962', 'Z7141'
Major depressive disorder	**ICD-9-CM**: '2962', '2963'<br>**ICD-10-CM**: 'F320', 'F321', 'F322', 'F323', 'F324', 'F325', 'F329', 'F330', 'F331', 'F332', 'F333', 'F3341', 'F3342', 'F339'
Anxiety disorder	**ICD-9-CM**: '300'<br>**ICD-10-CM**: 'F341', 'F4001', 'F4002', 'F4010', 'F40218', 'F40240', 'F40241', 'F408', 'F409', 'F410', 'F411', 'F418', 'F419', 'F42', 'F440', 'F441', 'F444', 'F446', 'F4481', 'F4489', 'F449', 'F450', 'F451', 'F4521', 'F4522', 'F458', 'F459', 'F481', 'F488', 'F489', 'F6811', 'F688', 'F99'

ICD-9-CM: International Classification of Diseases-9-Clinical Modification

ICD-10-CM: International Classification of Diseases-9-Clinical Modification

CPT-4: Current Procedural Technology-4

For the high-risk conditions during the 40-week pregnancy period, two diagnoses for the individual condition at least a day apart was required to be flagged as having that condition. For high-risk conditions at the month level, only one diagnosis in the respective month was required.

© 2023 Acharya M et al. *JAMA Network Open.*

eReferences

1. Patel SY, Mehrotra A, Huskamp HA, Uscher-Pines L, Ganguli I, Barnett ML. Variation in telemedicine use and outpatient care during the COVID-19 pandemic in the United States. *Health Affairs*. 2021;40(2):349-358. doi:10.1377/hlthaff.2020.01786
2. National Bureau of Economic Research. (2016). ICD-9-CM to and from ICD-10-CM and ICD-10-PCS crosswalk or general equivalence mappings.

© 2023 Acharya M et al. *JAMA Network Open.*
