# Supplementary material for: Trends in Telehealth Visits During Pregnancy, 2018 to 2021
Source: JAMA Netw Open. 2023 Apr 4;6(4):e236630. doi: 10.1001/jamanetworkopen.2023.6630 (PMC10074218; doi:10.1001/jamanetworkopen.2023.6630)
Supplement: Supplement 2. — Data Sharing Statement [file jamanetwopen-e236630-s002.pdf]

Data Sharing Statement

Acharya. Trends in Telehealth Visits During Pregnancy, 2018 to 2021. *JAMA Netw Open*. Published April 04, 2023. doi:10.1001/jamanetworkopen.2023.6630

Data
Data available: No

Additional Information
Explanation for why data not available: The dataset is proprietary and can be obtained from the data vendor.
